# High Goblet Cell Count Is Inversely Associated with Ploidy Abnormalities and Risk of Adenocarcinoma in Barrett’s Esophagus

**DOI:** 10.1371/journal.pone.0133403

**Published:** 2015-07-31

**Authors:** Amitabh Srivastava, Kevin L. Golden, Carissa A. Sanchez, Karen Liu, Pui Yee Fong, Xiaohong Li, David S. Cowan, Peter S. Rabinovitch, Brian J. Reid, Patricia L. Blount, Robert D. Odze

**Affiliations:** 1 Department of Pathology, Brigham & Women's Hospital, Boston, Massachusetts, United States of America; 2 Department of Human Biology, Fred Hutchinson Cancer Research Center, Seattle, Washington, United States of America; 3 Department of Public Health Sciences, Fred Hutchinson Cancer Research Center, Seattle, Washington, United States of America; 4 Department of Vaccine Infectious Disease Institute, Fred Hutchinson Cancer Research Center, Seattle, Washington, United States of America; 5 Department of Pathology, University of Washington, Seattle, Washington, United States of America; 6 Department of Medicine, University of Washington, Seattle, Washington, United States of America; 7 Department of Genome Sciences, University of Washington, Seattle, Washington, United States of America; University of Pennsylvania, UNITED STATES

## Abstract

**Purpose:**

Goblet cells may represent a potentially successful adaptive response to acid and bile by producing a thick mucous barrier that protects against cancer development in Barrett's esophagus (BE). The aim of this study was to determine the relationship between goblet cells (GC) and risk of progression to adenocarcinoma, and DNA content flow cytometric abnormalities, in BE patients.

**Experimental Design:**

Baseline mucosal biopsies (N=2988) from 213 patients, 32 of whom developed cancer during the follow up period, enrolled in a prospective dynamic cohort of BE patients were scored in a blinded fashion, for the total number (#) of GC, mean # of GC/crypt (GC density), # of crypts with ≥ 1 GC, and the proportion of crypts with ≥1 GC, in both dysplastic and non-dysplastic epithelium separately. The relationship between these four GC parameters and DNA content flow cytometric abnormalities and adenocarcinoma outcome was compared, after adjustment for age, gender, and BE segment length.

**Results:**

High GC parameters were inversely associated with DNA content flow cytometric abnormalities, such as aneuploidy, ploidy >2.7N, and an elevated 4N fraction > 6%, and with risk of adenocarcinoma. However, a Kaplan-Meier analysis showed that the total # of GC and the total # crypts with ≥1 GC were the only significant GC parameters (p<0.001 and 0.003, respectively).

**Conclusions:**

The results of this study show, for the first time, an inverse relationship between high GC counts and flow cytometric abnormalities and risk of adenocarcinoma in BE. Further studies are needed to determine if GC depleted foci within esophageal columnar mucosa are more prone to neoplastic progression or whether loss of GC occurs secondary to underlying genetic abnormalities.

## Introduction

In North America, Barrett’s esophagus (BE) is defined as columnar metaplasia, with goblet cells (GC), in the distal esophagus, although in some parts of the world, GC are not required for this diagnosis [1, 2]. There is abundant evidence to suggest that adenocarcinoma in BE develops via a columnar metaplasia/dysplasia/carcinoma pathogenic sequence [1,3,4]. However, recent population-based studies have reported that the risk of progression from BE to esophageal adenocarcinoma is substantially lower than earlier estimates, [5,6], and most patients with BE die of unrelated causes [1].

Columnar metaplasia occurs as a result of chemical/toxic damage secondary to reflux of gastric acid and bile into the distal esophagus, combined with release of inflammatory mediators [1]. Most patients with endoscopically recognizable columnar metaplasia have GC, and in the majority of patients with adenocarcinoma, the cancer arises in a background of neoplastic columnar mucosa with GC [7–10]. However, the precise role of GC in BE is uncertain [3,11,12]. It has been proposed that BE-associated metaplastic columnar epithelium represents a successful adaptation against the noxious effects of acid and bile [1,13–21]. This hypothesis has been based on the results of a number of discovery studies that have shown that Barrett’s-associated metaplastic epithelium secretes a thick adherent layer of mucus, as well as anions and bicarbonate, that decreases reflux-related injury [13,16]. Barrett's epithelium also possesses claudin-18 tight junctions that provides improved protection against acid permeation [17], and a crypt architecture that is believed to be tumor suppressive [18]. Metaplastic columnar cells have been shown to maintain intracellular pH following prolonged and repeated acid exposure [19]. One expression study, and another combined expression and proteomic study, concluded that Barrett's epithelium overexpresses genes involved in defense and repair of reflux-related injury [20,21]. All of these studies were appropriately designed for discovery research, but they involved small numbers of patients and did not directly evaluate the critical hypothesis of whether or not these mucosal adaptations modulate cancer development in BE and did not distinguish goblet from non-goblet columnar epithelium. We hypothesized that increasing numbers, density, and distribution of GC at baseline endoscopy protects against progression to adenocarcinoma and DNA content flow cytometric abnormalities (tetraploidy and aneuploidy), the latter of which are associated with an increased risk of progression. Therefore, the aims of this discovery study were to evaluate the association between the total quantity, density, and distribution of GC, with DNA content flow cytometry abnormalities, and the subsequent development of adenocarcinoma, in a cohort of BE patients.

## Materials and Methods

### 1. Patients

All patients in this study (N = 213) were part of the Seattle BE prospective surveillance program in which clinical, endoscopic, pathologic, and DNA content flow cytometric features were evaluated according to the previously described Seattle protocol [22, 23].

All patients included in this study had a baseline (index) endoscopy between 1995 and 1999 and at least one follow up endoscopy performed before 9/16/2009. All patients showed endoscopic evidence of columnar mucosa of the distal esophagus and were confirmed to have metaplastic columnar epithelium, with GC, in biopsies. Patients who had no evidence of adenocarcinoma at baseline endoscopy were placed under surveillance. Thus, all cancers in this study were categorized as “surveillance detected” esophageal adenocarcinoma.

In. total, 2988 biopsies at baseline endoscopy (mean per patient:14.0, range;1–64) were evaluated from 213 patients, who were followed for a mean of 90.4 months (range; 2.3–176 months). During follow up, 32/213 patients developed BE-associated adenocarcinoma defined as unequivocal evidence of glandular invasion into the mucosa, and/or submucosa. The study did not have sufficient numbers of patients to perform analyses of non-dysplastic biopsies in relationship to outcome for each diagnostic category (negative for dysplasia, low grade and high grade dysplasia) separately.

The Seattle Barrett’s Esophagus Study was approved by the Human Subjects Division of the University of Washington in 1983, and was renewed annually thereafter with reciprocity from the IRB of the Fred Hutchinson Cancer Research Center (FHCRC) from 1993–2001. Since 2001, the study has been approved annually by the IRB at the FHCRC with reciprocity from the Human Subjects Division of the University of Washington. All participants provided written informed consent for their clinical records to be used in this study.

### 2. Histologic methods

**Fig 1:**
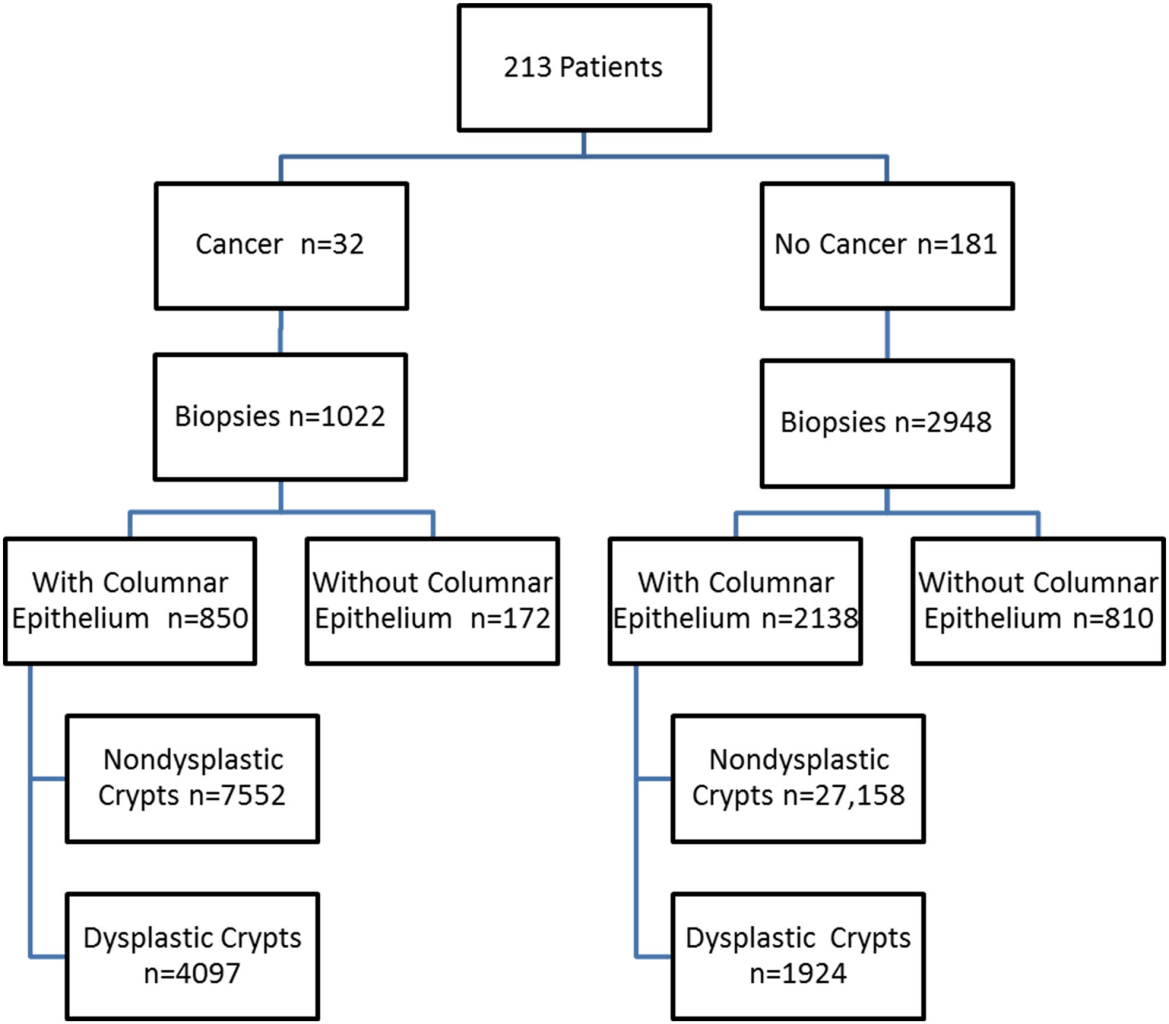
Distribution of Patient Samples. Distribution of biopsies with or without columnar epithelium, and number of dysplastic and non-dysplastic crypts in patients who did, or did not, progress to cancer. 172 biopsies without columnar epithelium from the cancer group and 810 biopsies of columnar epithelium from the non-cancer group were excluded from the statistical analysis.

Esophageal biopsies from the patient’s baseline endoscopy (N = 3970) were evaluated for the type of mucosa (squamous, oxyntic, cardiac, intestinal), and grade of dysplasia (negative, low grade or high grade) using previously published criteria [24]. Biopsies were evaluated by pathologists with the use of an Olympus BH-2 microscope without using any specialized morphometric technique. Biopsies with squamous or pure oxyntic epithelium, as well as biopsies with ulcers or without columnar mucosa, were excluded (Fig 1). The remaining biopsies (N = 2988) were systematically examined for GC counts as described further below. Three GI pathologists reached a consensus diagnosis for the highest grade of dysplasia in each biopsy, in a blinded manner, without knowledge of patient outcome.

One of the pathologists (AS) enumerated the total number of dysplastic and non-dysplastic crypts, separately, in each biopsy, and also scored each individual crypt as either negative, low-grade dysplasia (LGD), or high-grade dysplasia (HGD) as per previously published criteria [4]. When LGD and/or HGD was present in the biopsy, these areas were clearly marked with ink on the glass slide. The total number of GC, and the number of crypts with at least one GC, were then counted, in dysplastic and non-dysplastic crypts separately, by a second pathologist (KLG). Crypts that opened into the luminal surface and those that showed budding or branching at the base of the mucosa were scored as one crypt. In contrast, crypts that did not reach the luminal surface, but occupied the space between crypts that did, were counted as individual crypts. In this manner, we generated a total crypt count, a count for dysplastic and non-dysplastic crypts, and a count for the number of LGD and HGD crypts in biopsies with dysplasia. Using the GC counts mentioned above, the mean number (#) of GC per crypt (which represents the total #GC/total #crypts), also referred to as “GC density,” and the percentage of crypts with ≥1 GC (which represents the # crypts with ≥ 1 GC/#total crypts), were calculated for each biopsy. For per patient analyses, the mean values were calculated by averaging the GC values for all biopsies, obtained at the same endoscopy. All GC parameters were evaluated in baseline biopsies, in a blinded manner, without knowledge of patient outcome (cancer vs. no cancer).

### 3. Flow cytometry for DNA content

One fresh frozen biopsy was obtained from every two centimeters of BE and analysis of flow cytometric DNA histograms was performed as previously described [23,25]. Using a cutoff point identified by previous ROC analyses, 4N fractions above 6% were categorized as abnormal. Aneuploid fractions were further separated into <2.7N and >2.7N based on previously published criteria and methodology [26]. Similarly, 4N fractions were separated into <6%, 6–15% and >15% based on previously published criteria [26].

### 4. Statistical analysis

**Fig 2:**
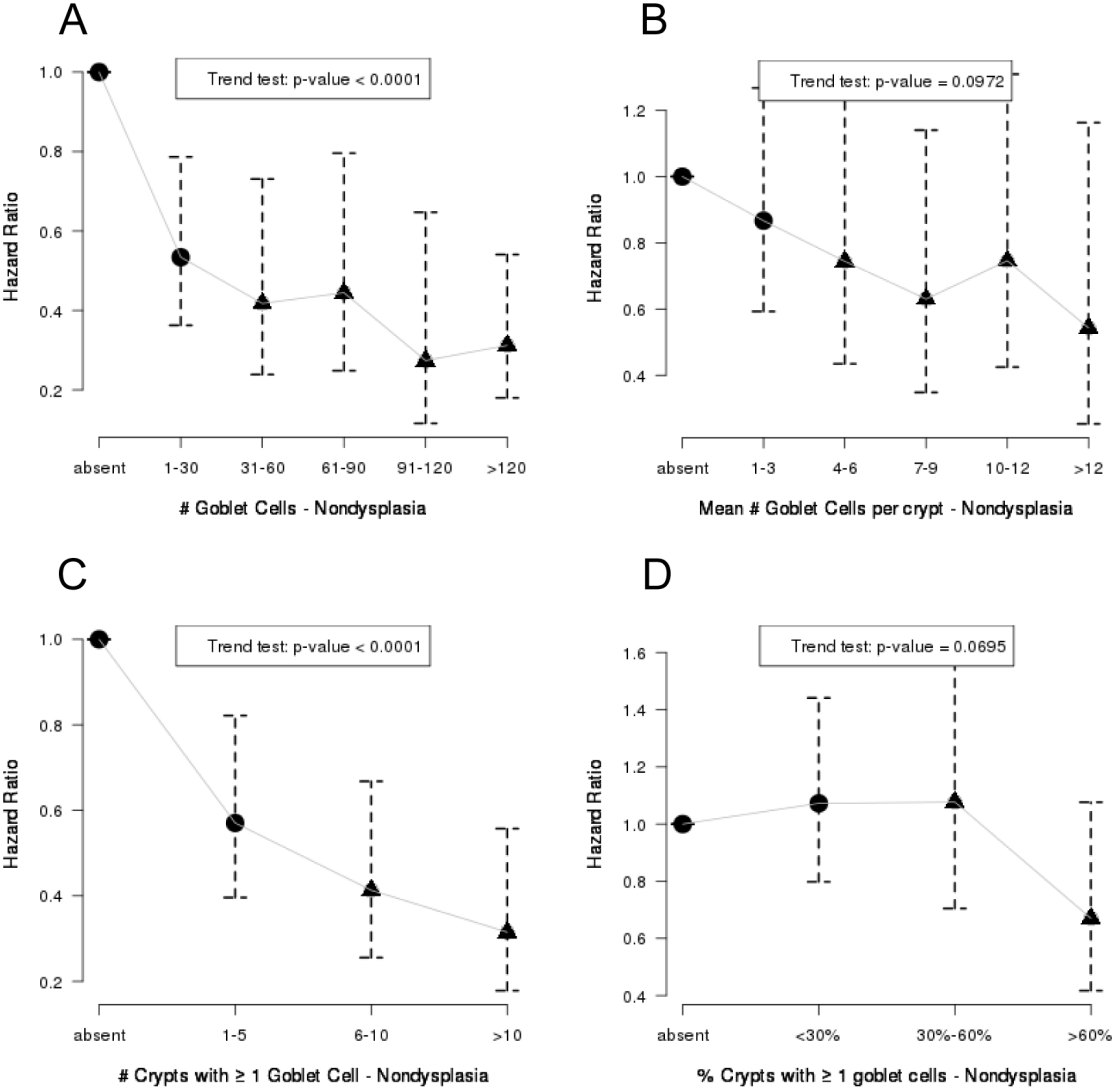
Goblet Cell Counts and Risk of Adenocarcinoma (Hazard Ratios). Association between # GC (A), mean #GC/crypt (B), # crypts with ≥ 1 GC (C) and proportion of crypts with ≥ 1GC (D), in non-dysplastic biopsies from patients with BE, and progression to adenocarcinoma was modeled using Cox regression on a per biopsy basis clustered by patient and adjusted for patient age, sex and BE length. The p-values were determined from the log rank test comparison between the biopsies from the categories indicated by a circle with those indicated by a triangle.

Cox regression analysis was used to assess the relationship between the # of GC, mean # GC/crypt, extent of GC (total # crypts with ≥1 GC, and proportion of crypts with ≥1 GC) and cancer outcome after adjustment for patient age, gender, and BE segment length. To perform the Cox regression analyses, multiple biopsies per patient were treated as a cluster in the likelihood calculation to ensure that we accounted for correlations between multiple biopsies from the same patient [27]. In this model, each patient may have more than one biopsy, so clusters with exchangeable correlation structure were used to account for correlated biopsies. Patients were binned into multiple categorical groups for each GC parameter for the hazard ratio analysis (Fig 2, six groups A and B, four groups in C and D). (S1 Table). Specific cutoff values used to establish the bins were chosen in order to ensure that there were roughly equivalent numbers of biopsies in each bin. The hazard ratios were calculated for each group using biopsies without GC as the reference group. Although the reference group represents biopsies without GC, all study patients had GC in biopsies obtained elsewhere in the esophageal columnar segment. The p-values for each plot were determined by a comparison of the log rank test between the categories indicated by a circle with those indicated by a triangle.

**Fig 3:**
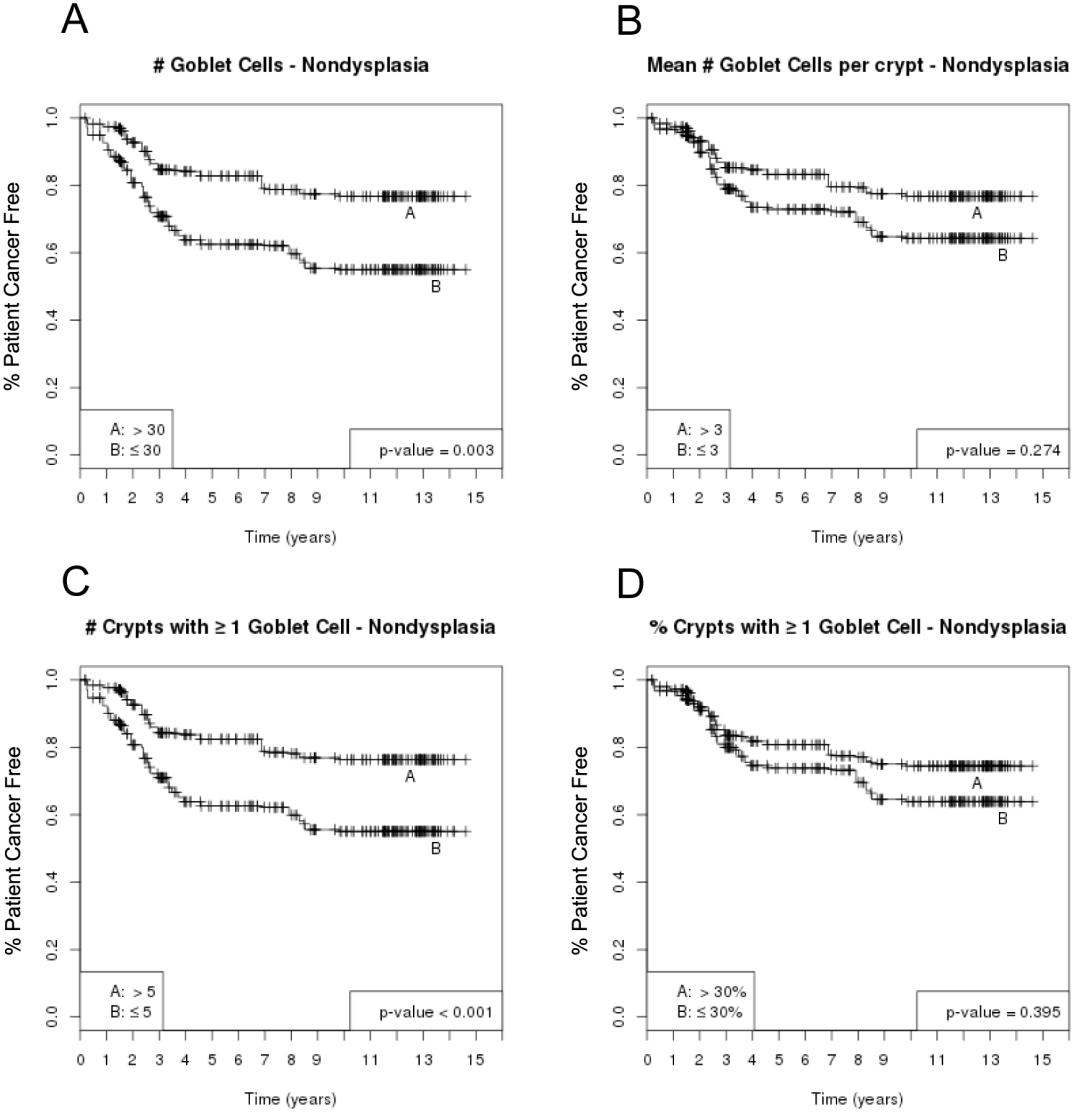
Goblet Cell Counts and Risk of Adenocarcinoma, Per Biopsy Analysis (Kaplan- Meier Curves). In a per biopsy analysis, the Kaplan-Meier curves show an inverse association between risk of adenocarcinoma and # GC (A), mean #GC/crypt (B), # crypts with ≥ 1 GC (C) and proportion of crypts with ≥ 1GC (D) in non-dysplastic biopsies from BE patients. P-values are from log-rank tests and have been adjusted for multiple comparison using the Benjamini & Hochberg method.

**Fig 4:**
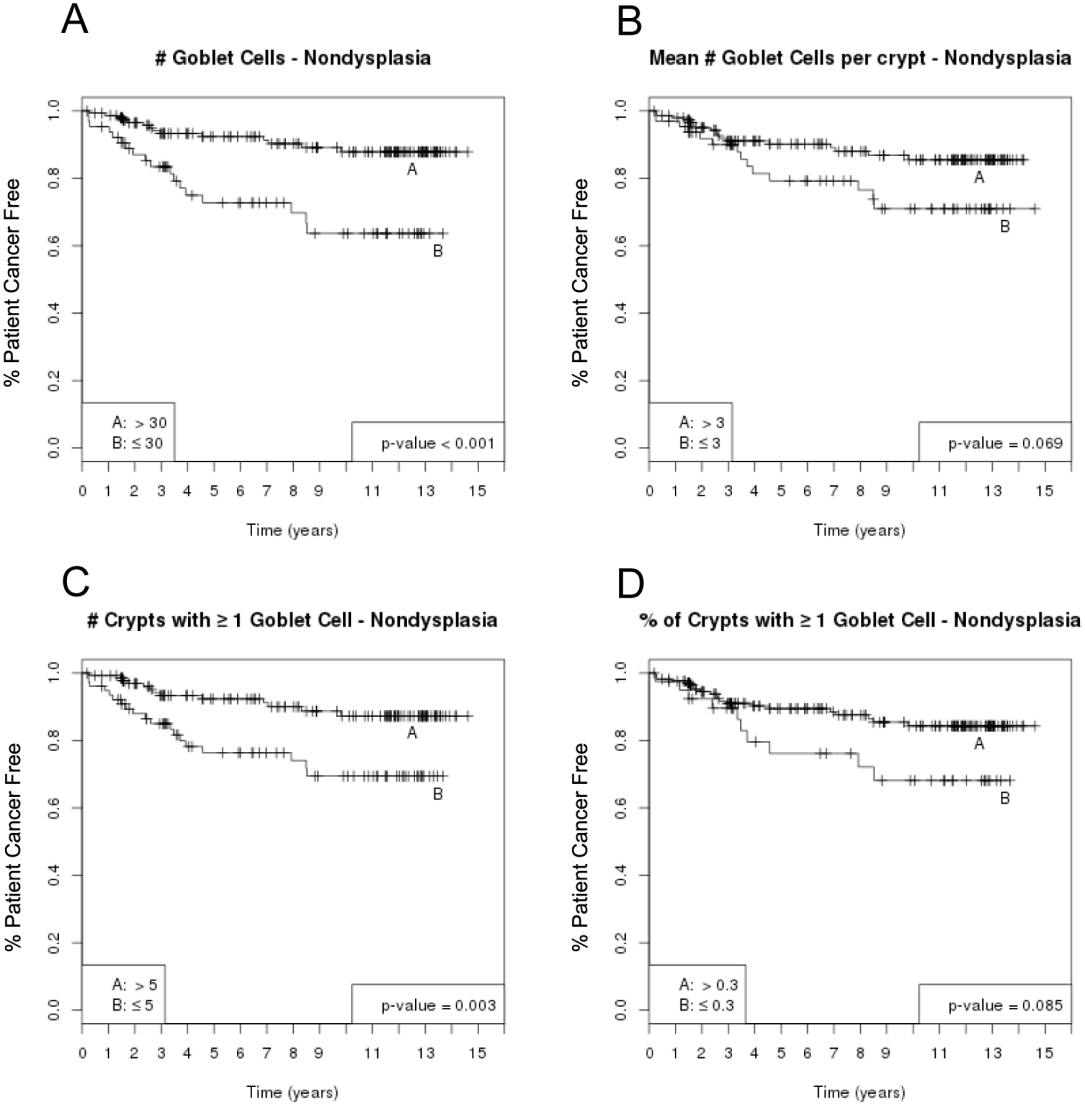
Goblet Cell Counts and Risk of Adenocarcinoma, Per Patient Analysis (Kaplan-Meier Curves). In a per patient analysis, the Kaplan-Meier curves show an inverse association between risk of adenocarcinoma and averaged GC parameters in non-dysplastic biopsies in each patient: # GC (A), mean #GC/crypt (B), # crypts with ≥ 1 GC (C) and proportion of crypts with ≥ 1GC (D). P-values are from log-rank tests and have been adjusted for multiple comparison using the Benjamini & Hochberg method.

Kaplan-Meier curves were used to assess the likelihood of developing adenocarcinoma in patients with low or high GC parameters. The cutoff values used for the KM curves (see Figs 3 and 4) were derived from the hazard ratio analysis above were not chosen to represent a rigorous diagnostic tool, but rather to explore a possible association between various GC parameters and the subsequent development of cancer. P values from log-rank tests have been adjusted for multiple comparisons using the Benjamini and Hochberg method [28]. Specifically, when multiple statistical tests are conducted, there is an elevated risk of false discovery due to the high number of statistical comparisons that are made. The Benjamini and Hochberg method is a commonly used method to adjust p-value thresholds for determination of significance in order that the overall false discovery rate would be controlled under a certain level. Many software programs offer standard procedures for using the Benjamini and Hochberg method. We used R-statistical package for implementing the method [27].

The equality of values in each GC parameter between aneuploidy (yes/no), or ploidy ≤2.7N and >2.7N, was compared using the Wilcoxon rank sum test. The trends across the tetraploid groups (4N ≤6%, >6–15%, and >15%) were tested using the non-parametric trend test. All comparisons were performed using the values for all biopsies combined, and for biopsies either with or without dysplasia separately. Generalized estimating equations clustered by patient were used to test the trend of GC parameter values related to the grade of dysplasia (S1 Fig). All analyses were implemented in R version 2.15.2 (2012-10-26). The p-values of the linear trend were determined with the Wald test.

## Results

### 1. Clinical characteristics

Table 1 summarizes the clinical and biopsy data of the patients in this study who either progressed (N = 32), or did not progress (N = 181), to cancer during follow up. Of the 2,988 biopsies with columnar epithelium evaluated in this study, 2,528 biopsies contained only non-dysplastic epithelium, 315 had only dysplastic epithelium, and 145 had a mixture of both non-dysplastic and dysplastic epithelium.

**Table 1:** Clinical data of patients who did or did not progress to cancer.

	Cancer	No Cancer	p-value
Parameter	N = 32 patients	N = 181 patients	
**Mean Age at baseline (years)**	65.6	62.5	0.16
**Gender (M:F ratio)**	4.3:1	3.8:1	0.77
**Mean BE length at baseline (cm) (min-max)**	8.22 (2–19)	5.26 (0–20)	<0.001
**Maximum diagnosis at baseline**			
* Negative for dysplasia*	5	144	
* Indefinite*	0	0	
* LGD*	3	27	
* HGD*	24	10	<0.001
**Mean # of biopsies/pt (min-max)**	26.6 (4–64)	11.8 (1–43)	<0.001
Mean # crypts*/biopsy (median, min-max)	13.7 (13, 0–47)	13.6 (12, 1–113)	0.73
**Mean # Nondysplastic crypts/biopsy (median, min-max)**	8.9 (8, 0–45)	12.7 (11, 0–113)	<0.001
**Mean # Dysplastic crypts/biopsy (median, min-max)**	4.8 (0, 0–47)	0.9 (0, (0–39)	<0.001
**Mean (median, min-max) follow-up in months**	41.3 (31, 2.9–118)	98.4 (107.1,2.3–176)	<0.001

* combined nondysplastic and dysplastic

### 2. Cross-sectional analysis of goblet cell parameters in non-dysplastic versus dysplastic epithelium

In a cross-sectional analysis of all baseline biopsies, all four GC parameters were evaluated in biopsies with only non-dysplastic epithelium (n = 2528), and the values were compared to biopsies with only LGD, (n = 161) and to biopsies with only HGD (n = 151). Biopsies that contained an admixture of both non-dysplastic and dysplastic epithelium, and those with foci of both low and high grade dysplasia, were excluded from this analysis (N = 148). As expected, there was a significantly higher number, density and distribution of GC in pure non-dysplastic epithelium compared to LGD epithelium, and similarly, in LGD epithelium compared to HGD epithelium (S1 Fig). The Wald test was used to determine the p-values for the linear trend for all four GC parameters analyzed, including total # GCs (p = 0.0009), mean # GC/crypt (GC density) (p = 0.0043), # crypts with ≥1 GC (p<0.0001) and proportion of crypts with ≥1 GC (p<0.0001).

### 3. Goblet cell parameters and risk of adenocarcinoma (per biopsy analysis)

The association between GC parameters and progression to adenocarcinoma was evaluated both on a per biopsy and a per patient level using Cox regression analysis. This analysis was performed using the GC values from all epithelium combined (both dysplastic and non-dysplastic), and only non-dysplastic and only dysplastic epithelium, separately. The specific data regarding analyses on all biopsies (combined non-dysplastic and dysplastic epithelium), biopsies with only dysplasia, and biopsies without dysplasia is outlined in (Table 2). A strong inverse association was present between all four GC parameters in *non-dysplastic epithelium* and risk of adenocarcinoma, even in patients who had dysplasia elsewhere in their esophagus (Table 2). The results were similarly significant, for all four GC parameters, when all 2988 biopsies (containing both dysplastic and non-dysplastic epithelium) were analyzed (p<0.0001 for all comparisons).

**Table 2:** Results of univariate analysis regarding goblet cell parameters in patients who did or did not progress to cancer.

Goblet Cell (GC) Parameters	All Biopsies			Biopsies with Dysplastic Crypts			Biopsies without Dysplastic Crypts		
	NC	C		NC	C		NC	C	
	*n = 2138*	*n = 850*	*P-value* ǂ	*n = 163*	*n = 297*	*P-value* ǂ	*n = 2048*	*n = 625*	*P-value* ǂ
**Number of Goblet Cells**									
* median (range)*	44 (0–1260)	12 (0–914)		6 (0–256)	3 (0–556)		46 (0–1260)	19 (0–914)	
* absent*	438	249	<0.0001	58	121	0.0420	412	159	0.0070
* 1–30*	493	283		56	122		465	191	
* 31–60*	258	87		18	22		248	70	
* 61–90*	199	60		17	12		185	50	
* 91–120*	145	32		5	9		139	23	
* >120*	605	139		9	10		594	128	
**Number of Crypts With ≥ One Goblet Cell**									
* median (range)*	7 (0–45)	3 (0–45)		2 (0–32)	1 (0–18)		7 (0–45)	4 (0–45)	
* absent*	436	250	<0.0001	60	126	0.0590	409	155	0.0160
* 1–5*	473	289		57	135		451	186	
* 6–10*	433	128		25	27		418	113	
* >10*	793	182		21	7		763	166	
**Number of Goblet Cells/crypt**									
* median (range)*	4.1 (0–106)	1.1 (0–49.75)		0.6 (0–22)	0.18 (0–44)		4.3 (0–106)	1.9 (0–56.4)	
* absent*	434	246	<0.0001	58	121	0.0420	412	159	0.0070
* >0–3*	518	312		52	129		482	197	
* 4–6*	323	94		28	24		302	82	
* 7–9*	245	64		12	8		236	55	
* 10–12*	166	45		5	6		166	41	
* >12*	448	84		8	8		445	87	
**Proportion of Crypts With ≥ Goblet Cell**									
* median (range)*	0.86 (0–1)	0.25 (0–1)		0.37 (0–1)	0.07 (0–1)		0.9 (0–1)	0.5 (0–1)	
* absent*	432	247	<0.0001	60	126	0.0590	409	155	0.0160
* 0–30%*	246	204		39	114		213	96	
* 30–60%*	203	106		12	26		192	82	
* >60%*	1,250	287		52	29		1,227	287	

NC = No cancer

C = Cancer

^ǂ^ p-value: test for the risk difference between absent/present of the feature

The inverse association between future risk of adenocarcinoma and each of the four GC parameters persisted after adjustment for gender, age, and BE segment length at baseline endoscopy. Higher values for total #GC and # crypts with ≥1GC were associated with a significantly lower risk of adenocarcinoma (Fig 2A and 2C), whereas those for mean GC/crypt and proportion of crypts with ≥1GC also showed an inverse association, but did not reach statistical significance after adjustment for multiple comparisons (Fig 2B and 2D), in the per biopsy analysis. We then combined categories of GC parameters, from this analysis, into low and high groups after observing the trends noted in (Fig 2) (denoted by solid circle and triangle, respectively). The Kaplan-Meier curves derived from this per biopsy analysis are shown in (Fig 3A, 3B, 3C and 3D). These results were reaffirmed in the per patient analysis shown in the next section below.

### 4. Goblet cell parameters and risk of adenocarcinoma (per patient analysis)

A per patient analysis using an average of each GC parameter in *non-dysplastic biopsies* at baseline endoscopy was then performed using the same cutoffs as described in the per biopsy analysis above. The association of all four GC parameters, in non-dysplastic epithelium, with cancer risk is shown in (Fig 4A, 4B, 4C and 4D). Once again, after adjusting for multiple comparisons, the Kaplan-Meier analysis indicated an inverse association between higher GC values in non-dysplastic epithelium and future risk of adenocarcinoma (Fig 4A, 4B, 4C and 4D). The association was statistically significant for total # GC and total # of crypts with ≥1 GC, (p<0.001 and p = 0.003, respectively). The mean GC/crypt and the proportion of crypts with ≥1 GC showed a similar trend, but the values did not reach statistical significance (p = 0.069 and p = 0.085, respectively).

### 5. Relationship between goblet cell parameters and DNA content flow cytometric abnormalities

**Fig 5:**
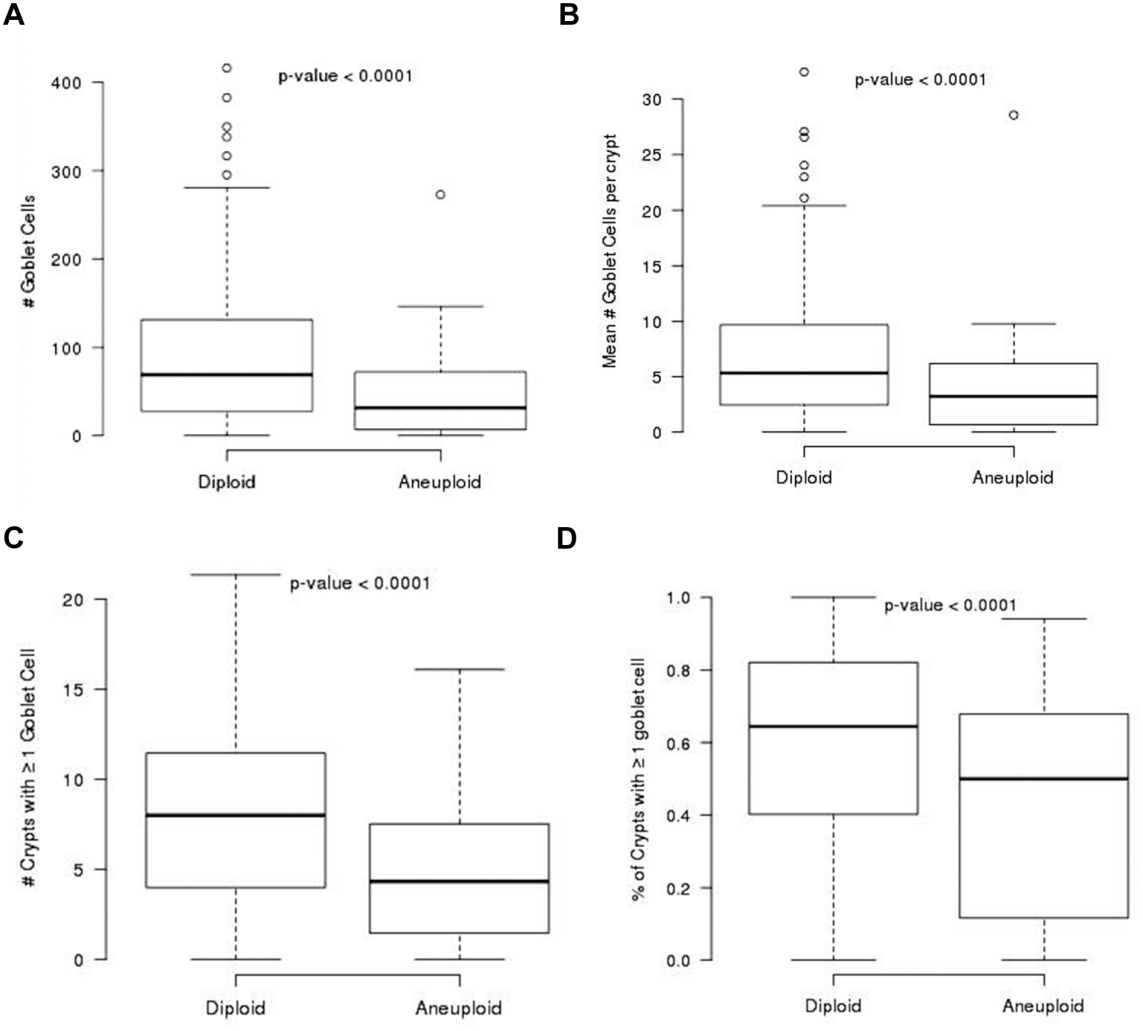
Goblet Cell Counts in Relationship to Aneuploidy. Non-dysplastic biopsies in patients with aneuploidy detected at baseline endoscopy have significantly lower # GC (A), mean #GC/crypt (B), # crypts with ≥ 1 GC (C) and proportion of crypts with ≥ 1GC (D) when compared to non-dysplastic biopsies in patients without aneuploidy at baseline endoscopy.

Similar to our outcome analysis, all analyses were performed, per patient, for the GC parameters in both dysplastic and non-dysplastic biopsies combined, and for only dysplastic or only non-dysplastic biopsies, separately. (Fig 5) shows the box plots for the relationship between all four GC parameters and aneuploidy (yes versus no) in *non-dysplastic epithelium*. To evaluate the relationship between the four GC parameters and DNA content flow cytometric abnormalities at baseline (cross-sectional data) and cancer outcome the values of each of the GC parameters were averaged among all the biopsies per patient. The association of the four GC parameters in all biopsies including non-dysplastic and dysplastic epithelium in relationship to abnormal ploidy per patient (≤2.7N vs >2.7N) and 4N fraction (<6% vs 6–15% vs >15%) are shown in (S1 Table). GC parameter values were significantly lower in patients who were aneuploid compared to those who were diploid regardless of whether the was analysis was performed using dysplastic (p values = 0.022, 0.0006, 0.022, and 0.0009 for total #GC, total # crypt with ≥1 GC, mean # GC/crypt, and proportion of crypts with ≥1 GC, respectively) or non-dysplastic crypts (p = 0.0041, 0.0042, 0.0094 and 0.0197, respectively, for similar comparisons).

### 6. Goblet cell parameters in progressors and nonprogressors

The distribution of coefficient of variation (CVs) of all the goblet cell parameters in progressors and nonprogressors showed high variation (greater than one) with the exception of one parameter, the proportion of crypts with ≥ 1GC (data not shown). These data underscore the heterogeneity in BE.

## Discussion

In this study, we evaluated the number (total # GC and mean # GC/crypt) and extent of distribution (# crypts with ≥1 GC and proportion of crypts with ≥1GC) of GC in BE patients in relationship to the development of adenocarcinoma and aneuploidy. We evaluated all four GC parameters in all biopsies (containing a sum of both dysplastic and non-dysplastic epithelium) as well as in only non-dysplastic epithelium, and only dysplastic epithelium, separately, in order to ensure that the results were not due simply to a loss of GC in dysplastic epithelium, as noted in the cross- sectional analysis portion of our study. The most significant, and novel, finding in our study was that a higher total number, density and extent of GC, in baseline non-dysplastic biopsies, were all negatively associated with future risk of adenocarcinoma. However, after adjusting for multiple comparisons, only the total # of GC and the total # crypts with ≥1 GC remained statistically significant in both our per biopsy and per patient analysis. All four GC parameters also showed an inverse association with flow cytometric DNA content abnormalities, such as aneuploidy, ploidy >2.7N, and an elevated 4N fraction > 6%.

Ours is the first study to evaluate the association between quantity, density, and extent of GC in baseline biopsies of patients with BE and subsequent progression to adenocarcinoma, and with DNA content flow cytometric abnormalities. Some retrospective cross-sectional studies of BE patients have shown that cancer occurs significantly more frequently in areas of low GC density (the latter measured on a semi quantitative scale) [29] while others have shown that areas of mucosa with high GC density do not correlate with foci of high-grade dysplasia/cancer [30]. A prior cross- sectional study, by Liu et al, suggested that DNA content abnormalities occur with equal frequency in non-dysplastic columnar epithelium either with, or without, GC and that these alterations are independent of the extent of GC in the esophagus [11]. Several other studies have evaluated molecular abnormalities in patients with esophageal columnar metaplasia, either with or without GC [31,32]. In these studies, molecular abnormalities were detected in both non-goblet and goblet containing columnar epithelium but mutations were not correlated with the degree of GC metaplasia. Goblet cells secrete a type of mucin (MUC2) that inhibits the development of colon cancer in mouse models. MUC2 deficient mice develop adenocarcinomas [33] and when crossed with APC deficient mice, the result is accelerated tumor development and metastasis [34,35]. Based on the design and results of our study, we do not advocate use of GC parameters as a clinical diagnostic marker of cancer risk. However, our data provides a better understanding of the possible role of GC in mucosal defense by utilizing well-designed cohort of BE patients. This knowledge may help provide insight into novel strategies and targets for adenocarcinoma prevention, as well as a better understanding of critical elements that may contribute to risk of progression.

Previous studies by our group, as well as others, have shown that neoplastic progression in BE occurs as a result of genomic instability and subsequent clonal expansion of selected cell populations that have a growth advantage [1,23,26]. Our finding of an inverse relationship between high GC parameters in non-dysplastic epithelium and DNA content flow cytometric abnormalities supports a potential role of GC in neoplastic progression in BE. After adjusting for multiple comparisons, the total # GC and total # crypts with ≥1 GC were, statistically, the most significant GC parameters inversely associated with future risk of adenocarcinoma, on both a per biopsy and per patient analysis.

Our study design did not allow us to determine whether GC depleted foci within esophageal columnar mucosa are more prone to neoplastic progression, or whether loss of GC in non-dysplastic epithelium represents a phenomenon that occurs secondary to underlying genetic abnormalities. We also could not determine whether GC differentiation is affected by the chemical composition of the refluxate. Our data showing a negative association between GC and risk of adenocarcinoma, and aneuploidy, in only non-dysplastic biopsies, and in non-dysplastic biopsies from patients who had dysplasia elsewhere in their esophagus, argues strongly against the possibility that our results were simply due to the fact that dysplastic epithelium is relatively GC depleted compared to non-dysplastic epithelium. This data also suggests that GC differentiation diminishes first in non-dysplastic epithelium and then subsequently in dysplastic epithelium.

Mucous is produced from both non-goblet (mucinous) columnar cells and GC, but the relative contribution of each of these types of cells to the production of an adherent surface mucous layer in BE patients, and the protective effects of each type of mucin, have never been evaluated. Dixon et al, in a study of four patients in 2001, noted the presence of an adherent mucous layer in columnar-lined esophagus and proposed that the mucous layer is likely to offer protection against refluxed acid and bile [13]. Most interestingly, a transmission electron microscopic study of BE patients, by Levine et al in 1989, showed a strong association between DNA content flow cytometric abnormalities, such as tetraploidy and aneuploidy, and disruption (depletion, physical alteration) of intracellular organelles involved in mucous biosynthesis [15]. Disruption of certain biological pathways of cell differentiation, such as the Notch Pathway, may affect the phenotype of BE and the risk for neoplastic progression [36].

There are limitations to our study. Firstly, our study did not contain enough outcomes of interest in each category (negative for dysplasia, LGD or HGD) in order to perform analyses confined to each subcategory of BE patients. However, our goal was to explore the association between goblet cells and adenocarcinoma and not to develop a novel clinical risk prediction model. Our results regarding an inverse relationship between total number of GC and number of crypts with ≥1 GC and risk of adenocarcinoma and DNA content abnormalities, persisted when only non-dysplastic epithelium was used in the analysis. Another possible limitation was that all measurements were made by pathologists using a microscope on standard two-dimensional tissue specimens, without the use of sophisticated stereological or morphometric methods. Unfortunately, to the best of our knowledge, there are no morphometric methods available that can reliably distinguish grades of dysplasia in mucosal biopsy specimens, and none that can reliably distinguish true goblet cells from goblet cell mimics, such as distended foveolar cells (pseudogoblet cells).

There is debate, worldwide, regarding the requirement to identify GC in mucosal biopsies of the esophagus in order to establish a diagnosis of BE [12]. Several retrospective and cross-sectional studies have documented an increased risk of neoplasia (dysplastic and/or cancer) in patients with esophageal columnar metaplasia, but without GC [37]. In fact, in one study by Takubo et al, 56.6% of small adenocarcinomas excised by endoscopic mucosal resection developed in areas of mucosa completely devoid of GC [38]. However, other studies, such as one by Bhat et al, has shown that the risk of neoplastic progression in patients with columnar metaplasia without GC is substantially lower than in patients with columnar metplasia with GC (0.07% incidence per year vs 0.38%) [6]. Unfortunately, our study was not designed to test, or challenge, the North American BE criteria, since all of the patients in our study had GC in their esophageal columnar mucosa at baseline endoscopy.

In summary, the results of this study suggest that high GC counts have an inverse relationship with the presence of DNA content flow cytometric abnormalities and risk of adenocarcinoma, in patients with BE. Loss of GC may represent a biological mechanism that enhances development of adenocarcinoma, or may be a secondary phenomenon due to other factors such as genetic abnormalities that also are responsible for neoplastic progression. Further longitudinal studies are needed to determine the nature of the association between GC and adenocarcinoma.

## Supporting Information

S1 Fig Goblet Cell Counts in Dysplastic Epithelium. Loss of goblet cell differentiation occurs with onset of dysplasia. The # GC (A), mean #GC/crypt (B), # crypts with ≥ 1 GC (C) and proportion of crypts with ≥ 1GC (D) are all significantly reduced when only non-dysplastic biopsies were compared to biopsies with only low grade dysplasia (LGD) and high grade dysplasia (HGD). Mean and standard error are shown in the figure. The p-values were calculated from a trend test using Generalized Estimating Equations, clustered by patient.
(TIF)

S1 Table Summary of goblet cell parameters in relationship to flow cytometric abnormalities. Summary of goblet cell parameters in relationship to flow cytometric abnormalities such as aneuploidy, ploidy, and 4N fraction. These values are separated into all patients (N = 213), patients with any biopsies with dysplasia (N = 64), and patients with any non-dysplastic biopsies (N = 212). The data points are expressed as "yes", or "no" for aneuploidy, ≤ or > 2.7N for ploidy, and <6%, 6–15%, and >50% for N fraction.
(DOCX)
