# Supplementary figures and images for: High Goblet Cell Count Is Inversely Associated with Ploidy Abnormalities and Risk of Adenocarcinoma in Barrett’s Esophagus
Source: PLoS One. 2015 Jul 31;10(7):e0133403. doi: 10.1371/journal.pone.0133403 (PMC4521918; doi:10.1371/journal.pone.0133403)

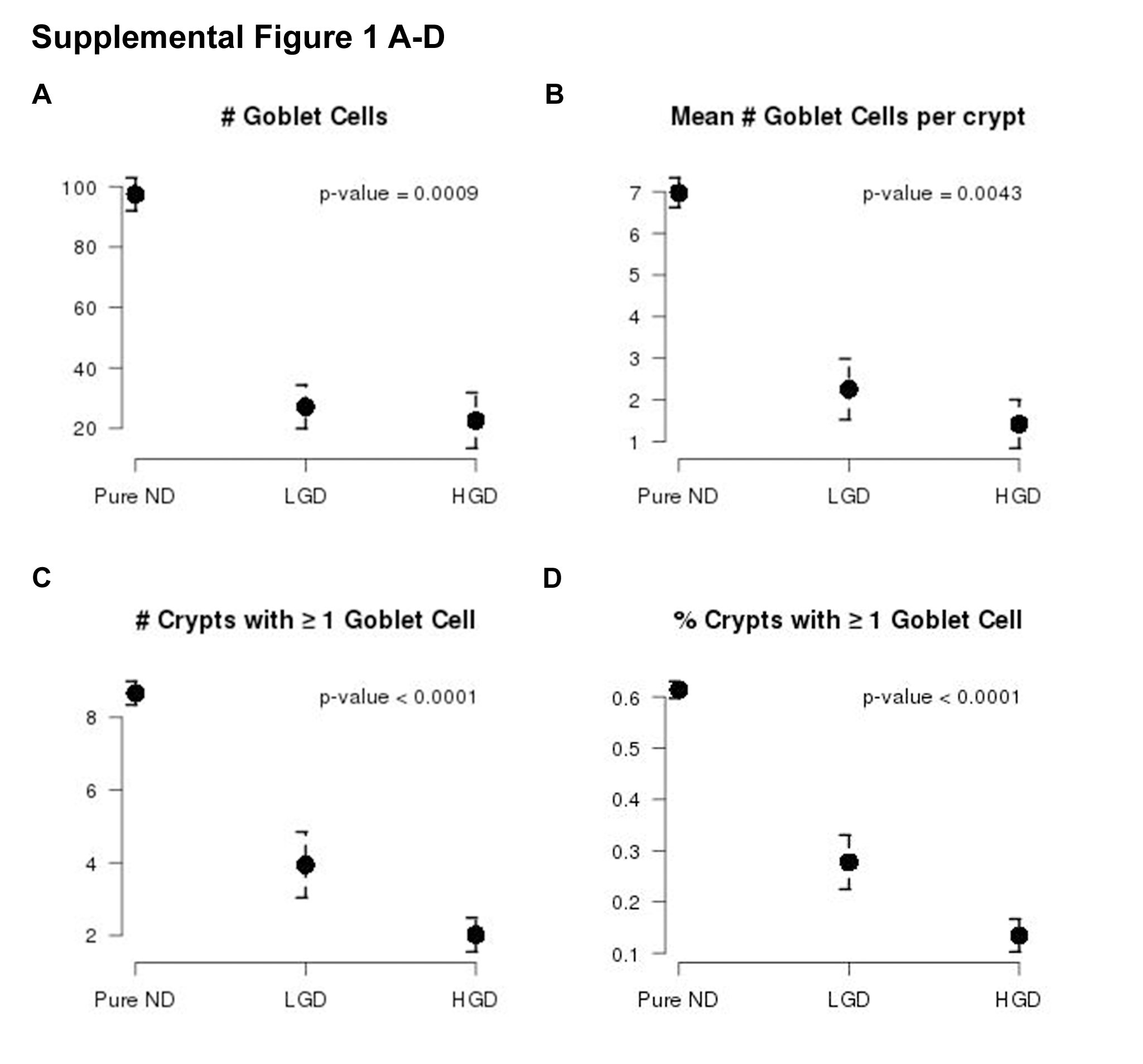

Supplement: S1 Fig — Loss of goblet cell differentiation occurs with onset of dysplasia. The # GC (A), mean #GC/crypt (B), # crypts with ≥ 1 GC (C) and proportion of crypts with ≥ 1GC (D) are all significantly reduced when only non-dysplastic biopsies were compared to biopsies with only low grade dysplasia (LGD) and high grade dysplasia (HGD). Mean and standard error are shown in the figure. The p-values were calculated from a trend test using Generalized Estimating Equations, clustered by patient. (TIF) [file pone.0133403.s001.tif]
