# Supplementary material for: High Goblet Cell Count Is Inversely Associated with Ploidy Abnormalities and Risk of Adenocarcinoma in Barrett’s Esophagus
Source: PLoS One. 2015 Jul 31;10(7):e0133403. doi: 10.1371/journal.pone.0133403 (PMC4521918; doi:10.1371/journal.pone.0133403)
Supplement: S1 Table — Summary of goblet cell parameters in relationship to flow cytometric abnormalities such as aneuploidy, ploidy, and 4N fraction. These values are separated into all patients (N = 213), patients with any biopsies with dysplasia (N = 64), and patients with any non-dysplastic biopsies (N = 212). The data points are expressed as "yes", or "no" for aneuploidy, ≤ or > 2.7N for ploidy, and <6%, 6–15%, and >50% for N fraction. (DOCX) [file pone.0133403.s002.docx]

**Supplementary Table 1: Summary of goblet cell parameters in relationship to flow cytometric abnormalities**

|  | | | | | | | | | | | | |  |  |  |
| --- | --- | --- | --- | --- | --- | --- | --- | --- | --- | --- | --- | --- | --- | --- | --- |
|  |  |  |  | | |  | Aneuploid | |  | Ploidy |  |  | 4N |  |  |
|  |  |  |  | | |  | mean |  |  | mean |  |  | mean |  |  |
|  |  |  |  | | | Yes | No | p-value | <=2.7N | >2.7N | P-value | <6% | 6-15% | >15% | P-value |
| **All patients ( N= 213)** | | | | | | N=26 | N=187 |  | N=192 | N=21 |  | N=187 | N=21 | N=5 |  |
|  | **Number of GC** | | | |  | 43.0554 | 91.8803 | 0.0016 | 90.5549 | 43.5487 | 0.0061 | 91.7123 | 46.0954 | 36.5716 | 0.0020 |
|  | **Number of Crypts With ≥ One GC** | | | | | 4.8540 | 8.0948 | 0.0010 | 8.0218 | 4.7498 | 0.0021 | 8.0433 | 5.3663 | 4.6260 | 0.0070 |
|  | **Number of GC per crypt** | | | | | 3.4520 | 6.9757 | 0.0019 | 6.8818 | 3.4717 | 0.0054 | 6.9934 | 3.3021 | 3.4207 | 0.0010 |
|  | **Proportion of Crypts With ≥ One GC (%)** | | | | | 0.3846 | 0.5931 | 0.0004 | 0.5880 | 0.3811 | 0.0013 | 0.5910 | 0.3957 | 0.4137 | 0.0020 |
| **# patients with any biopsies with dysplasia ( N=64 )** | | | | | | N=20 | N=44 |  | N=46 | N=18 |  | N=43 | N=16 | N=5 |  |
|  | **Number of GC** | | |  | | 12.5130 | 31.4483 | 0.0216 | 30.1853 | 13.6367 | 0.0831 | 30.5527 | 17.8792 | 6.8307 | 0.0480 |
|  | **Number of Crypts With ≥ One GC** | | | | | 1.5003 | 4.9815 | 0.0006 | 4.7758 | 1.6392 | 0.0050 | 4.6464 | 2.8433 | 0.7807 | 0.0020 |
|  | **Number of GC per crypt** | | | | | 1.3829 | 3.2343 | 0.0216 | 3.1249 | 1.4566 | 0.0521 | 3.3828 | 1.2507 | 0.8991 | 0.0040 |
|  | **Proportion of Crypts With ≥ One GC (%)** | | | | | 0.1733 | 0.5046 | 0.0009 | 0.4853 | 0.1859 | 0.0058 | 0.5004 | 0.2219 | 0.1205 | 0.0010 |
| **# patients with any non-dysplastic biopsies ( N= 212)** | | | | | | N=25 | N=187 |  | N=192 | N=20 |  | N=187 | N=20 | N=5 |  |
|  | **Number of Goblet Cells** | | | | | 49.0019 | 91.7428 | 0.0041 | 90.4071 | 51.1393 | 0.0144 | 91.2948 | 55.5090 | 39.7301 | 0.0080 |
|  | **Number of Crypts With ≥ One GC** | | | | | 5.1017 | 7.9868 | 0.0042 | 7.9154 | 5.0658 | 0.0094 | 7.9133 | 5.7879 | 5.1077 | 0.0370 |
|  | **Number of GC per crypt** | | | | | 4.4688 | 7.0131 | 0.0094 | 6.9134 | 4.7897 | 0.0329 | 7.0134 | 4.4875 | 4.3844 | 0.0150 |
|  | **Proportion of Crypts With ≥ One GC (%)** | | | | | 0.4457 | 0.5948 | 0.0197 | 0.5894 | 0.4606 | 0.0665 | 0.5904 | 0.4670 | 0.5248 | 0.0790 |
| GC = Goblet cell | | |  | | |  |  |  |  |  |  |  |  |  |  |
